# Bacterium-like Particles from *Corynebacterium pseudodiphtheriticum* as Mucosal Adjuvant for the Development of Pneumococcal Vaccines

**DOI:** 10.3390/vaccines12040412

**Published:** 2024-04-12

**Authors:** Ramiro Ortiz Moyano, Fernanda Raya Tonetti, Mariano Elean, Yoshiya Imamura, Kohtaro Fukuyama, Yoshihito Suda, Vyacheslav Melnikov, Alexander Suvorov, María Guadalupe Vizoso-Pinto, Haruki Kitazawa, Julio Villena

**Affiliations:** 1 Laboratory of Immunobiotechnology, Reference Centre for Lactobacilli (CERELA-CONICET), San Miguel de Tucumán 4000, Argentina; rortiz@cerela.org.ar (R.O.M.); frayatonetti@gmail.com (F.R.T.); melean@cerela.org.ar (M.E.); 2 Food and Feed Immunology Group, Laboratory of Animal Food Function, Graduate School of Agricultural Science, Tohoku University, Sendai 981-8555, Japan; yoshiya.imamura.p8@dc.tohoku.ac.jp (Y.I.); kotaro.fukuyama.p8@dc.tohoku.ac.jp (K.F.); 3 Livestock Immunology Unit, International Education and Research Center for Food and Agricultural Immunology (CFAI), Graduate School of Agricultural Science, Tohoku University, Sendai 981-8555, Japan; 4 Department of Food, Agriculture and Environment, Miyagi University, Sendai 980-8572, Japan; suda@myu.ac.jp; 5 Gabrichevsky Research Institute for Epidemiology and Microbiology, 125212 Moscow, Russia; 6 Federal State Budgetary Scientific Institution “Institute of Experimental Medicine”, 197022 Saint Petersburg, Russia; alexander_suvorov1@hotmail.com; 7 Infection Biology Laboratory, Instituto Superior de Investigaciones Biológicas (INSIBIO), CONICET-UNT, San Miguel de Tucumán 4000, Argentina; mgvizoso@fm.unt.edu.ar

**Keywords:** *Corynebacterium pseudodiphtheriticum*, bacterium-like particles, mucosal adjuvant, respiratory infection, pneumococcal vaccines

## Abstract

Previously, it was shown that intranasally (i.n.) administered *Corynebacterium pseudodiphtheriticum* 090104 (Cp) or CP-derived bacterium-like particles (BLPs) improve the immunogenicity of the pneumococcal conjugate vaccine (PCV). This work aimed to deepen the characterization of the adjuvant properties of Cp and CP-derived BLPs for their use in the development of pneumococcal vaccines. The ability of Cp and CP-derived BLPs to improve both the humoral and cellular specific immune responses induced by i.n. administered polysaccharide-based commercial pneumococcal vaccine (Pneumovax 23^®^) and the chimeric recombinant PSPF (PsaA-Spr1875-PspA-FliC) protein was evaluated, as well as the protection against *Streptococcus pneumoniae* infection in infant mice. Additionally, whether the immunization protocols, including Cp and CP-derived BLPs, together with the pneumococcal vaccines can enhance the resistance to secondary pneumococcal pneumonia induced after inflammatory lung damage mediated by the activation of Toll-like receptor 3 (TLR3) was assessed. The results showed that both Cp and CP-derived BLPs increased the immunogenicity and protection induced by two pneumococcal vaccines administered through the nasal route. Of note, the nasal priming with the PSPF T-dependent antigen co-administered with Cp or CP-derived BLPs efficiently stimulated humoral and cellular immunity and increased the resistance to primary and secondary pneumococcal infections. The CP-derived BLPs presented a stronger effect than live bacteria. Given safety concerns associated with live bacterium administration, especially in high-risk populations, such as infants, the elderly, and immunocompromised patients, BLPs emerge as an attractive mucosal adjuvant to improve the host response to pneumococcal infections and to enhance the vaccines already in the market or in development.

## 1. Introduction

*Streptococcus pneumoniae* causes several diseases such as pneumonia, meningitis, otitis, and sinusitis, which in some cases may require hospitalization or may even cause death. This bacterium especially threatens children under 2 years and the elderly [1]. One of the aspects that makes it difficult to control the spreading of this pathogen is asymptomatic carriers, who have pneumococcus in their throat or nose, and transmit the bacteria to close contacts via aerosols. Usually, susceptible individuals develop pneumonia, for which the immune response is critical for clearing the bacteria and preventing sepsis [2,3]. Two types of vaccines are available on the market to combat *S. pneumoniae*: pneumococcal conjugate vaccines (PCV13, PCV15, and PCV20) and pneumococcal polysaccharide vaccines (PPSV23). They do not cover all the existing serotypes and, therefore, they confer an incomplete protection, resulting in people having a pneumococcal disease more than once [1]. Further, the so-called “serotype replacement effect” appears when a vaccinated population is susceptible to serotypes not included in the vaccine formulation [4]. Thus, there is a need for serotype-independent vaccines, which elicit an efficient immune response against *S. pneumoniae*. Additionally, two important shortcomings of these vaccines are their low immunological memory in high-risk populations and their low efficiency in generating mucosal responses [4].

In our previous studies, we tested an experimental vaccine made out of a chimeric protein consisting of a fragment of *Salmonella enterica* subsp. *enterica* serovar Typhimurium flagellin FliC and three fragments of the following *S. pneumoniae* surface proteins: the pneumococcal surface adhesion A protein (PsaA), the pneumococcal surface protein A (PspA), and the surface protein Spr1875 [5]. The resulting chimeric protein PSPF (PsaA-Spr1875-PspA-FliC) of 560 amino acids was produced using heterologous expression in *Escherichia coli*. We combined the chimeric vaccine with heat-killed *Lacticaseibacillus rhamnosus* CRL1505 or its cell wall to improve the efficacy of the vaccine and administered it i.n. to stimulate the immune response at the mucosal level. Both the cell wall and the heat-killed bacteria were effective in eliciting specific IgA and IgG responses in the respiratory tract [5]. Lu et al. [6] evaluated *Lactococcus lactis*-derived BLPs to formulate a PspA-BLP vaccine comprising PspA proteins from two distinct families, specifically, PspA2 from family 1 and PspA4 from family 2. The vaccine formulation utilized the BLP adjuvant not only for its adjuvant properties but also as the carrier. Intranasal immunization with the PspA-BLP vaccine demonstrated effective induction of both PspA2- and PspA4-specific IgG in the serum and PspA2- and PspA4-specific IgA in the mucosa. Furthermore, the analysis of serum antibodies revealed strong binding to intact bacteria, including pneumococcal strains expressing PspA from clades 1 to 5. Importantly, the PspA-BLP vaccine exhibited significant protective efficacy against challenges from pneumococcal strains belonging to different PspA families. These results suggest that innocuous bacteria with immunomodulatory properties or their cellular fractions can be used as efficient mucosal adjuvants.

In line with our research, it was demonstrated that commensals, such as *Clostridium ramosum* [7] or *Mycobacterium bovis* [3,8], can act as vectors to deliver antigens as well as adjuvants. *Corynebacterium pseudodiphtheriticum* is a member of the normal human nasopharynx microbiota whose role has been related to the improvement of protection against respiratory infections [9]. Previously, we demonstrated that C. *pseudodiphtheriticum* strain 090104 (*Cp 090104*) i.n. administered to mice enhanced the resistance to respiratory syncytial virus (RSV) [10], *S. pneumoniae* [11], and *Klebsiella pneumoniae* [12]. The protective ability of the *Cp 090104* strain against the different pathogenic microorganisms was associated to its capacity to regulate the innate immune responses in the respiratory tract. It was observed that *Cp 090104* stimulated lung dendritic cells (DCs) [9] and alveolar macrophages, enhancing their ability to synthesize interferon (IFN)-β and IFN-γ after the stimulation of Toll-like receptor (TLR3) [10] or TLR2 [11]. Interestingly, bacterium-like particles (BLPs) from *Cp 090104*, obtained through acid-heat treatment, were also effective to modulate DCs and alveolar macrophages when intranasally administered to infant mice [10,11].

Recently, we evaluated the capacity of *Cp 090104* and CP-derived BLPs, when co-administered with the pneumococcal vaccine Prevenar^®^13 via the nasal route, to improve the protection of mice against pneumococcal infection [13]. It was found that the *Cp 090104* strain and the CP-derived BLPs stimulated the respiratory innate immune system, serving as adjuvants to enhance the specific antibody response against the vaccine, which contains 13 pneumococcal capsular polysaccharides all conjugated to the CRM197 carrier protein. This immunomodulatory effect of *Cp 090104* and the CP-derived BLPs correlated with significantly lower lung and blood counts of the pathogen in the mice challenged with *S. pneumoniae* serotypes 6B or 19F [13]. In this work, we aimed to advance the characterization of the adjuvant properties of *Cp 090104* and the CP-derived BLPs and their capacities to enhance mucosal and systemic responses to pneumococcal vaccines. For this purpose, we used a polysaccharide-based commercial pneumococcal vaccine (Pneumovax 23^®^) and the chimeric recombinant PSPF protein [5] and evaluated the ability of *Cp 090104* and the CP-derived BLPs to improve both the humoral and cellular specific immune responses and the protection against *S. pneumoniae* infection in infant mice. Furthermore, we evaluated for the first time whether the immunization protocols, including the *Cp 090104* strain and the CP-derived BLPs, together with the pneumococcal vaccines can enhance the resistance to secondary pneumococcal pneumonia induced after inflammatory lung damage mediated by the activation of the viral pattern recognition receptor TLR3.

## 2. Materials and Methods

### 2.1. C. pseudodiphteriticum Culture and Obtention of BLPs

*Cp 090104*, a respiratory commensal bacterium, was grown in trypticase soy broth at 37 °C for 18 h. Bacterial suspensions were meticulously prepared through a process involving the washing of bacteria with sterile phosphate-buffered saline (PBS, 0.01 M, pH 7.2) at 3000× *g* for a span of 10 min. Subsequently, the bacteria were suspended in sterile PBS [11]. The production of BLPs ensued through a heat-chemical treatment, and was executed in adherence to previously delineated protocols [13]. Briefly, the *Cp 090104* strain underwent a thorough wash with sterile water (13,000× *g* for 10 min) and was then immersed in 0.1 M HCl before being boiled 45 min in a water bath. The resultant BLPs were subjected to three rounds of washing (PBS, pH 7.2), preserved at −20 °C, and suspended in an appropriate volume in order to reach a concentration equivalent to 10^9^ UFC/mL.

### 2.2. Animals, Immunizations, and Pneumococcal Infections

For investigations involving primary pneumococcal infection, female 3-week-old Swiss albino mice were employed in our experimental setup because of their susceptibility to pneumococcal infection [5,11]. The exploration of secondary pneumococcal infection involved a model incorporating poly(I:C), a synthetic analog of double-stranded RNA (dsRNA) recognized using pattern recognition receptors (PRRs), such as TLR3 and retinoic acid-inducible gene I (RIG-I), and *S. pneumoniae* superinfection, and was conducted in female 3-week-old BALB/c mice because of the susceptibility of this mouse strain to TLR3-mediated lung damage [14]. The animal subjects were sourced from the enclosed colony maintained at CERELA-CONICET in San Miguel de Tucumán, Argentina. Both mice strains were accommodated in plastic cages under controlled room temperature conditions with a 12 h light–dark cycle. Throughout the experiments, the mice were provided with a conventional balanced diet. This investigation adhered to the guidelines outlined in the Guide for the Care and Use of Laboratory Animals and the Guidelines for Animal Experimentation of CERELA, following the BI-OT-CRL/19 protocol. All measures were taken to minimize any potential discomfort or distress experienced by the animals.

The doses of *Cp 090104* (10^8^ CFU) and the CP-derived BLPs (10^8^ particles) were selected in previous studies and prepared from an overnight culture [10,11]. CFU/mL were determined using serial dilutions plated onto MRS agar. For particle counts a Thoma chamber was used.

In the initial series of experiments, 3-week-old Swiss albino infant mice were subjected to nasal administration of *Cp 090104* (10^8^ cells) or the CP-derived BLPs (10^8^ particles) for a consecutive 5-day period. One day after the final stimulation (day 6), the mice underwent nasal challenge with *S. pneumoniae* serotype 6B (10^5^ CFU/mouse). For this, the mice were lightly anesthetized, and then we administered a dropwise solution of 50 μL PBS containing 250 μg poly(I:C) (equivalent to 10 mg/kg body weight) through the nares. Control mice were given 50 μL of PBS.

Secondly, in the next set of experiments, different groups of Swiss albino mice (3-week-old) received 20 μg of the polysaccharide-based commercial pneumococcal vaccine (Pneumovax 23^®^, PPV, MSD S.R.L., Buenos Aires, Argentina), PPV together with 10^8^ cells of *Cp 090104*, PPV and 10^8^ BLPs, 20 μg of PSPF [5], PSPF and 10^8^ cells of *Cp 090104*, or PSPF and 10^8^ BLPs on days 0, 14, and 28. The different vaccine formulations suspended in 25 µL PBS were administered to animals intranasally. On day 33, different samples were taken for the evaluation of adaptive immunity or for challenging with *S. pneumoniae*. The infectious challenge was performed through the nasal route by administering 10^6^ CFU *S. pneumoniae* serotypes 6B or 19F in 25 µL PBS.

In the third set of experiments, BALB/c infant mice (3-week-old) were immunized via nasal route with 20 μg of PSPF, PSPF together with 10^8^ cells of *Cp 090104*, or PSPF and 10^8^ BLPs on days 0, 14, and 28. Five days after the last immunization (day 33), the mice were i.n. stimulated with poly(I:C) for 3 consecutive days, and 5 days after the last poly(I:C) administration, the mice were infected with *S. pneumoniae.* The infection with the respiratory pathogen was performed through the nasal route by administering 25 µL PBS containing 10^4^ CFU of *S. pneumoniae* serotypes 6B or 19F.

### 2.3. Antibody Quantification

Specific anti-*S. pneumoniae*, anti-PPV, and anti-PSPF IgM, IgA, and IgG antibodies were measured using ELISA (enzyme-linked immunosorbent assay) in serum and broncho-alveolar lavages (BALs). One μg of PPV or PSPF, or 10^4^ heat-killed cells of *S. pneumoniae* were coated onto wells overnight at 4 °C. Albumin was used to block the plates. The samples were diluted (serum 1:20; BAL 1:2) and incubated for 1 h at 37 °C. Antibodies against mouse IgG, IgM, or IgA conjugated with peroxidase (1:500) (Sigma-Aldrich, San Luis, MO, USA) were added and incubated for 1 h at 37 °C. Concentrations of antibodies were assessed using referencing standard curves constructed with known quantities of the corresponding mouse immunoglobulins (Sigma-Aldrich, San Luis, MO, USA).

### 2.4. Infection Challenge Experiments and Pneumococcal Cell Counts

For infection experiments, *S. pneumoniae* serotype 6B and 19F were cultured on blood agar at 37 °C for 18 h, followed by a passage onto Todd Hewitt broth (THB, Oxoid, Cambridge, UK) overnight at 37 °C. Pneumococci were harvested 3600 g for 10 min and washed with sterile PBS. The challenge of animals was performed via the nasal route with 10^4^, 10^5^, or 10^6^ CFU of pneumococci per mouse, as described above. *S. pneumoniae* counts in lung and blood were determined on day 2 or 7 post-infection [11].

After euthanasia, lungs were excised, weighed, and homogenized in sterile peptone water. The respective homogenates were diluted and plated onto blood agar for bacterial counts (CFU log//g lung). Bacteremia was reported as either negative or positive.

### 2.5. Lung Tissue Injury Markers

To assess the augmented permeability of the bronchoalveolar–capillarity barrier, the determination of albumin content in cell-free BAL was conducted. Furthermore, the quantification of lactate dehydrogenase (LDH) activity served as an indicator of overall cytotoxicity [9,10].

### 2.6. Cytokine Concentrations

BAL samples, collected as detailed in prior studies [9,10], were preserved at −70 °C. The concentrations of tumor necrosis factor (TNF)-α, interferon (IFN)-γ, and interleukin 4 (IL-4) in both serum and BAL were determined using ELISA, in accordance with the manufacturer’s guidelines (R&D Systems, Minneapolis, MN, USA).

### 2.7. Statistical Analysis

The experiments were conducted in triplicate, with each experimental group comprising three mice at each designated time point (*n* = 9 for each assessed parameter). The findings were presented as mean ± standard deviation (SD), and statistical analysis involved the use of the Student’s *t*-test to discern significant differences between groups, with significance set at *p* < 0.05. Additionally, analysis of variance among multiple groups was carried out using the one-way ANOVA.

## 3. Results

### 3.1. Cp 090104 and CP-Derived BLPs Improve the Adaptive Immune Response against S. pneumoniae in Infant Mice

**Figure 1:**
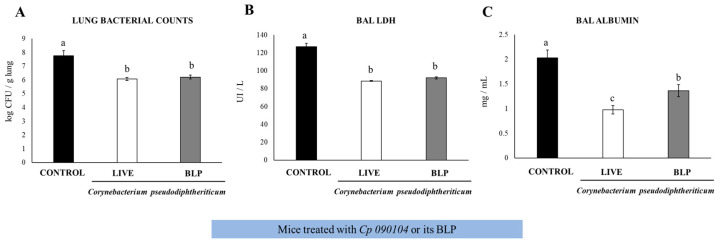
Impact of intranasally administered *Cp 090104* or CP-derived BLPs on the resistance of infant mice to *S. pneumoniae* infection. Three-week-old Swiss albino infant mice were subjected to nasal treatment with *Cp* 090104 (10^8^ cells) or CP-derived BLPs (10^8^ particles) for a continuous 5-day period. On the sixth day, the mice faced nasal challenge with *S. pneumoniae* serotype 6B (10^5^ CFU/mouse). The evaluation of lung pneumococcal cell counts (**A**), along with the determination of lactate dehydrogenase (LDH) (**B**), and albumin (**C**) levels in bronchoalveolar lavage (BAL) samples, took place on day 7 post-infection. The experiments were conducted in triplicate, involving three mice per group (*n* = 9). The results are expressed as mean ± SD. Statistical differences between the groups are denoted by letters (*p* < 0.05).

We previously demonstrated the ability of the *Cp 010904* strain and CP-derived BLPs to beneficially modulate the respiratory innate immunity against pneumococcal infection by evaluating immunological parameters at 2 days post-infection [10,11]. Here, we evaluated the resistance to the infection at day 7 and the adaptive immune response. We i.n. primed 3-week-old Swiss mice with live *Cp 090104* or CP-derived BLPs for five consecutive days. Then, we infected the mice i.n. with *S. pneumoniae* serotype 6B and followed the mice for signs of disease for 7 days until euthanasia. Control mice had bacterial loads of 5.6 × 10^7^ CFU/g lung and both treatments reduced the burden of bacterial colonization of lungs by two log units (Figure 1A).

In the control mice, *S. pneumoniae* spread and was detected in blood in contrast to the *Cp 090104*- and BLPs-treated mice that had negative hemocultures. Accordingly, the evaluation of the tissular injury parameters in BALs showed that both treatments reduced lung injury as measured using BAL albumin concentrations and LDH activity (Figure 1B,C). Notably, the viable *Cp 090104* was significantly better (*p* < 0.05) than BLPs in reducing the BAL albumin concentration (Figure 1C).

**Figure 2:**
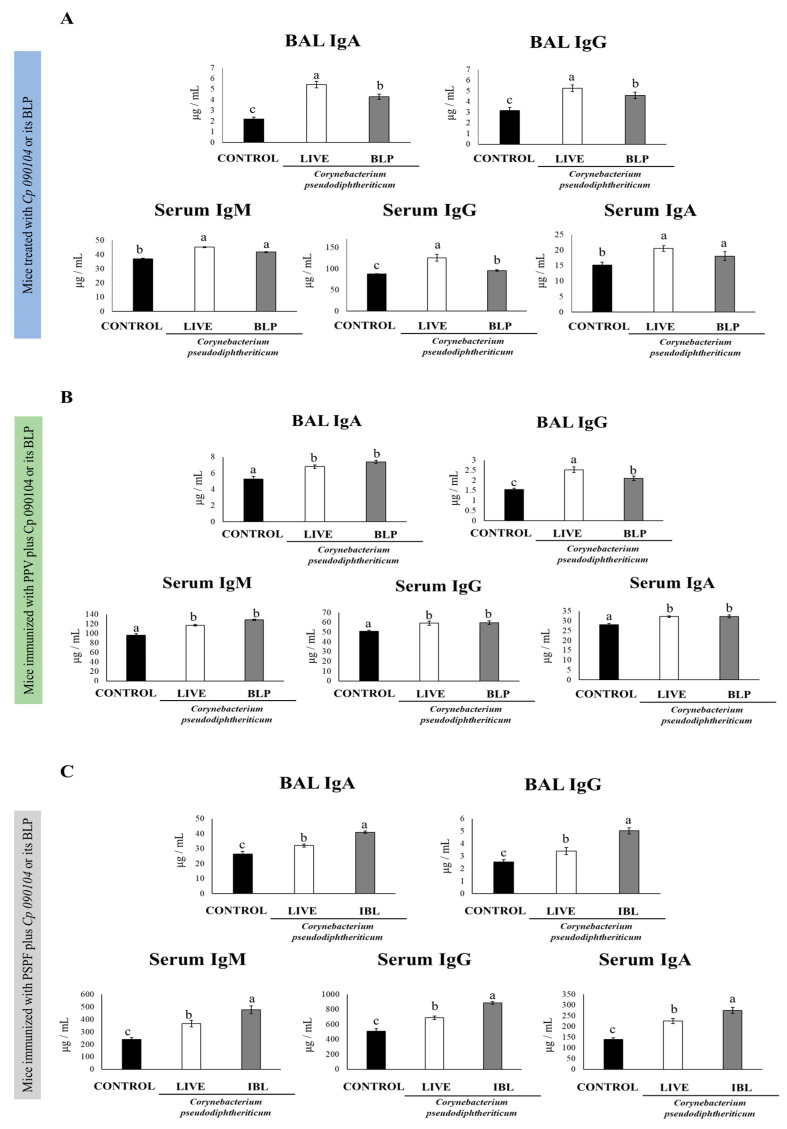
Impact of intranasally administered *Cp 090104* or CP-derived BLPs on (**A**) the humoral response of infant mice to *S. pneumoniae* infection, (**B**) immunization with the polysaccharide pneumococcal vaccine (PPV), and (**C**) immunization with the recombinant PsaA-Spr1875-PspA-FliC chimeric protein. ELISA was employed to assess the concentrations of pneumococcal-specific IgA and IgG antibodies in BALs, and IgM, IgG, and IgA in serum. The experiments were conducted in triplicate, each involving three mice per group (*n* = 9). Results are presented as mean ± SD. Statistical differences between the groups are denoted by letters (*p* < 0.05).

We next evaluated the humoral and cellular adaptive immune responses at day 7 post-infection. The nasal administration of live *Cp 090104* or the CP-derived BLPs improved the production of specific immunoglobulins against *S. pneumoniae* both locally and systemically. While both live bacteria and particles significantly increased (*p* < 0.05) the production of anti-pneumococcal antibodies in the respiratory tract, the effect of live *Cp 090104* was more pronounced on both IgA and IgG production compared to the BLPs (Figure 2A).

**Figure 3:**
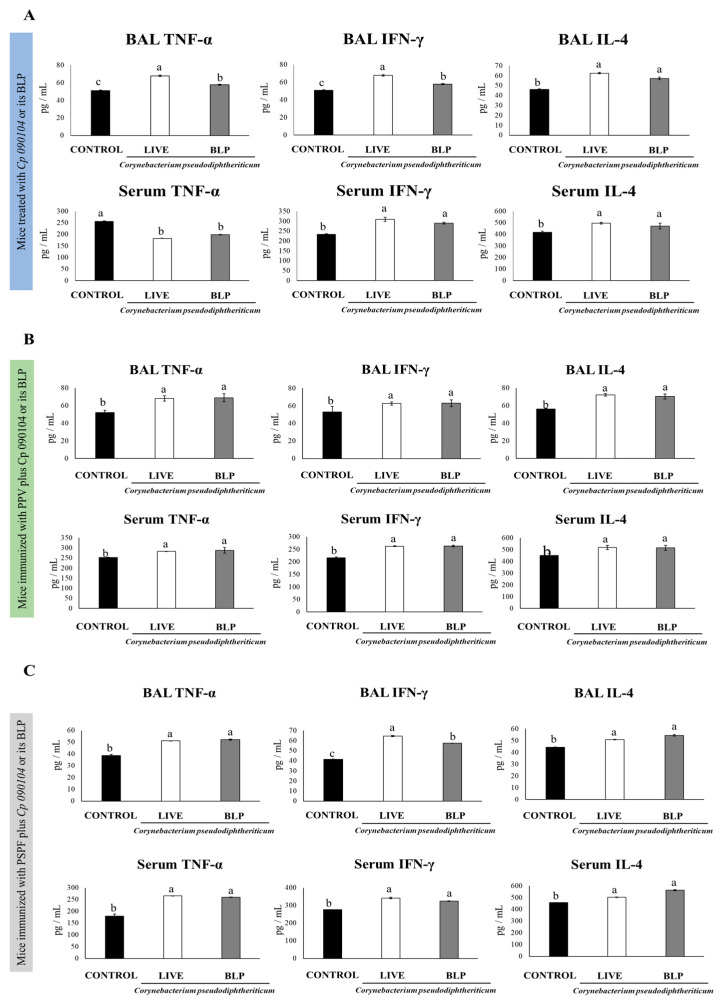
Impact of intranasally administered *Cp 090104* or CP-derived BLPs on cytokine levels in BAL and serum to evaluate the response of infant mice to (**A**) *S. pneumoniae* infection, (**B**) immunization with the polysaccharide pneumococcal vaccine (PPV), and (**C**) immunization with the recombinant PsaA-Spr1875-PspA-FliC chimeric protein (PSPF). Three-week-old Swiss albino infant mice underwent nasal treatments. Seven days post-pneumococcal challenge, serum and bronchoalveolar lavage (BAL) samples were collected for the determination of TNF-α, IFN-γ, and IL-4. The experiments were conducted in triplicate, each involving three mice per group (*n* = 9). Results are presented as mean ± SD. Statistical differences between the groups are highlighted by letters (*p* < 0.05).

The different types of specific immunoglobulins against *S. pneumoniae*, IgM, IgG, and IgA were enhanced in sera of the mice receiving the nasal pretreatment with *Cp 090104* or CP-derived BLPs before pneumococcal infection in comparison to the control mice (Figure 2A). Only in the serum anti-pneumococcal IgG, live *Cp 090104* induced significantly higher levels than the CP-derived BLPs. We also evaluated the changes in the concentration of cytokines produced by T cells at this time point post-infection: IL-4, TNF-α, and IFN-γ in BAL and serum. The three cytokines were significantly (*p* < 0.05) enhanced in BAL in the pre-treatment with BLPs or live bacteria compared with the control mice (Figure 3A). The same was mirrored for IL-4 and IFN-γ in serum. Surprisingly, the levels of TNF-α in serum were lower in pre-treated animals than in the control mice (Figure 3A). Notably, live bacteria were more efficient in inducing an increase in IFN-γ and TNF-α in the respiratory tract compared to BLPs.

### 3.2. Cp 090104 and CP-Derived BLPs Improve the Adaptive Immune Response Induced by a Commercial Pneumococcal Polysaccharide Vaccine

Considering the ability of *Cp 090104* and CP-derived BLPs to improve both the innate and adaptive immune responses, we hypothesized that both treatments could serve as mucosal adjuvants when intranasally administered with pneumococcal antigens. Thus, to study the adjuvant potential of *Cp 090104*, we immunized 3-week-old Swiss mice with a commercial pneumococcal polysaccharide vaccine (PPV, a T cell-independent vaccine) following an immunization schedule of three doses separated by 14 days from each other. The mice were immunized intranasally with PPV plus *Cp 090104* or the CP-derived BLPs. On day 33, we evaluated the specific antibody response in the blood and BAL (Figure 2B). Serum IgM, IgG, and IgA levels were enhanced (*p* < 0.05) in the mice that received *Cp 090104* or CP-derived BLPs compared to the mice receiving the PPV only (Figure 2B). Further, there were no significant differences between treatments, with live bacteria and BLPs being equally effective in enhancing the humoral systemic immune response. Similarly, IgA and IgG levels in BALs were also higher in the mice immunized with BLPs and live bacteria in comparison with the mice immunized with the vaccine only. At the respiratory mucosa, live *Cp 090104* induced significantly (*p* < 0.05) higher IgG levels than the BLPs (Figure 2B).

We aimed to test whether the induction of an enhanced immune response by the PPV plus *Cp 090104* or the CP-derived BLPs correlated with a higher protection against pneumococci. On day 33, after the last boosting with the PPV, we infected the immunized mice with *S. pneumoniae* (6B or 19F). Forty-eight hours after infection, animals were euthanized to evaluate the susceptibility to the infection. We first quantified the degree of lung invasion by determining the pneumococcal cell counts in lung tissue.

We measured the cytokines IL-4, IFN-γ, and TNF-α in BAL and serum to assess the activation of the specific cellular immune response after the pneumococcal challenge. The mice that received the PPV together with live bacteria or the CP-derived BLPs had higher levels of the three cytokines in both BAL and sera compared with the mice that received the PPV alone after the infection with serotype 6B (Figure 3B). BLPs and live *Cp 090104* did not differ significantly in the modulation of these three cytokines (Figure 3B).

**Figure 4:**
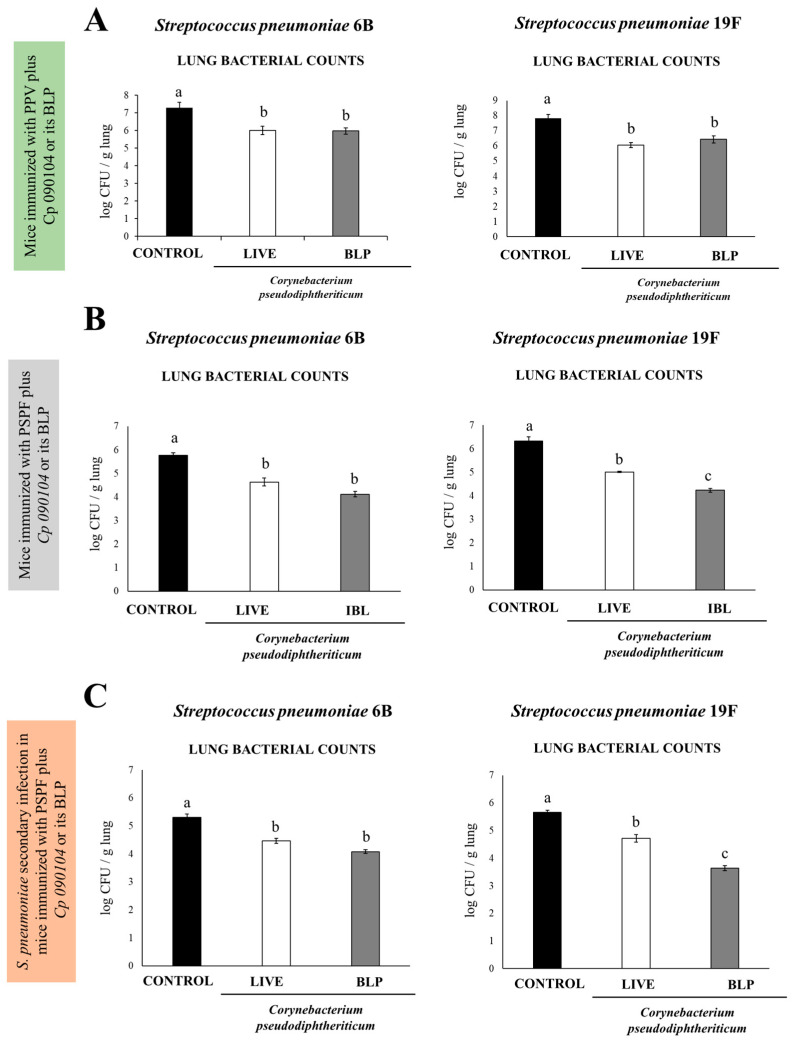
Examining the protective effect against *S. pneumoniae* infection on infant mice conferred by (**A**) immunization with the polysaccharide pneumococcal vaccine (PPV) combined with either *Cp 090104* or CP-derived BLPs and (**B**) immunization with the recombinant PsaA-Spr1875-PspA-FliC (PSPF) chimeric protein combined with either *Cp 090104* or CP-derived BLPs. (**C**) Protection against secondary *S. pneumoniae* infection of infant mice after the immunization with the recombinant PsaA-Spr1875-PspA-FliC chimeric protein combined with either *Cp 090104* or CP-derived BLPs. Three-week-old Swiss albino infant mice underwent nasal immunizations with PPV (20 μg) alone either PSPF (20 μg) alone or combined with *Cp 090104* (10^8^ cells) or CP-derived BLPs (10^8^ particles) on days 0, 14, and 28. On day 33, the mice were challenged nasally with *S. pneumoniae* serotypes 6B (10^6^ CFU/mouse) or 19F (10^6^ CFU/mouse). Lung pneumococcal cell counts were conducted on day 2 post-infection. The experiments were performed in triplicate, each involving three mice per group (*n* = 9). The results are presented as mean ± SD, and significant differences between the groups were denoted by letters (*p* < 0.05).

The outcomes illustrated in Figure 4A indicate a reduction of approximately 1.5 logs in bacterial counts for the mice administered *Cp 090104* or CP-derived BLPs in comparison to animals immunized solely with PPV. We also determined whether *S. pneumoniae* reached the bloodstream by performing hemocultures, which were negative in the mice immunized with PPV plus *Cp 090104* or CP-derived BLPs but positive for animals immunized with PPV only.

Similar results were obtained when the infection was performed with *S. pneumoniae* serotype 19F (Figure 4A).

### 3.3. C. pseudodiphteriticum 090104 and CP-Derived BLPs Improve the Adaptive Immune Response Induced by the Experimental Chimeric Recombinant Protein Vaccine PSPF

We followed the same immunization protocol as in the previous experiments but changed the type of vaccine and used in this case a chimeric recombinant vaccine (PSPF) [5] administered alone or in combination with *Cp 090104* or CP-derived BLPs. On day 33, the mice were euthanized, and we analyzed the PSPF-specific antibodies present in serum and BAL using ELISA (Figure 2C). Specific IgA and IgG levels were higher in the mice immunized with the PSPF administered with *Cp 090104* or CP-derived BLPs than in the mice that only received PSPF. Furthermore, the effect of the adjuvants tested with this vaccine was much stronger than that observed using the polysaccharide vaccine. In fact, BLPs were more effective in inducing IgA and IgG in the BAL than the live bacteria in this set of experiments using the PSPF (Figure 3C). In serum, the same tendency was observed for IgM, IgA, and IgG. PSPF delivered with *Cp 090104* or CP-derived BLPs stimulated significantly (*p* < 0.05) more elevated levels of antibodies than PSPF alone, and BLPs were better than live bacteria in inducing specific antibodies (Figure 3C).

We also studied the protection conferred by the experimental chimeric recombinant vaccine alone or administered with BLPs and live *Cp 090104* to the challenges with *S. pneumoniae* serotypes 6B or 19F. For comparison purposes, we used the same immunization, infection, and sampling schema explained before. Two days after the challenge (day 33) bacterial counts were determined in lung tissue. For both serotypes, the immunization with PSPF added with BLPs or live *Cp 090104* conferred better protection than the PSPF alone, as reflected by the different bacterial burdens found in the lungs (Figure 4C). When comparing the BLPs and live bacteria administered with PSPF, BLPs resulted in lower bacterial counts in lungs for the serotype 19F. In all cases, the hemocultures of the vaccinated mice after the challenge were negative, in contrast to the non-vaccinated mice. The outcomes were comparable when the infection was conducted using *S. pneumoniae* serotype 19F (Figure 4B).

When cytokines levels were determined in BAL and serum samples, it was observed that both *Cp 090104* and CP-derived BLPs were comparable in terms of the increase in TNF-α and IL-4 elicited at the respiratory mucosa and in blood after the challenge with serotype 6B (Figure 3C). Similarly, both treatments induced higher BAL and serum IFN-γ when compared with the mice immunized only with PSPF. Notably, the mice receiving PSPF and BLPs showed higher BAL IFN-γ levels than animals vaccinated with PSPF plus live *Cp* (Figure 3C).

### 3.4. Immunization with PSPF and C. pseudodiphteriticum 090104 or CP-Derived BLPs Improves Resistance to Secondary Pneumococcal Infection Induced after Viral Inflammation

In the end, we studied whether the immunization protocol including the PSPF and the mucosal adjuvants *Cp 090104* and BLPs could influence the resistance of the mice to secondary pneumococcal pneumonia produced after the inflammatory lung damage triggered by TLR3 activation. Thus, to model a respiratory viral infection preceding a bacterial one, we inoculated infant BALB/c mice i.n. on day 33 after the immunization, with poly(I:C) for three successive days. After five days, the mice were challenged with *S. pneumoniae* serotypes 6B or 19F. The resistance to the infection and the immune response were studied 48 h after the pneumococcal challenge (Figure 4C). The lung bacterial counts in animals inoculated with serotype 6B increased by 1 log when compared to the initial inoculum for the control mice but remained at the same level in animals who previously received live *Cp 090104* or the BLPs. For the more virulent 19F serotype, the protective effect of *Cp 090104* or BLPs was superior (*p* < 0.05) to that observed for serotype 6B. In this case, BLPs were significantly better (*p* < 0.05) than live bacteria, reducing the bacterial counts by 2 log when compared to the control (Figure 4C). The hemocultures were negative for all the experimental groups.

**Figure 5:**
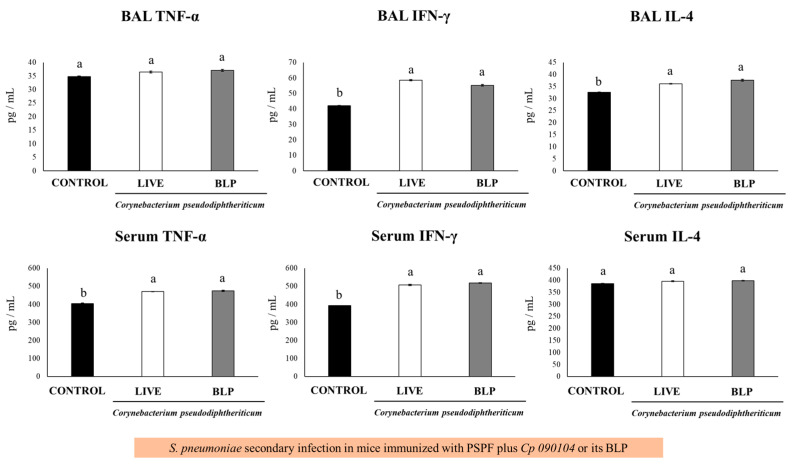
Immune response to secondary *S. pneumoniae* infection following immunization with PSPF in conjunction with Cp 090104 or CP-derived BLPs in 3-week-old BALB/c infant mice. The immunization protocol involved administration of PSPF (20 μg) or PSPF (20 μg) in combination with Cp 090104 (10^8^ cells) or CP-derived BLPs (10^8^ particles) on days 0, 14, and 28. Subsequently, on day 33, the mice were intranasally stimulated with poly(I:C) for three consecutive days, and five days after the final poly(I:C) administration, the mice underwent nasal challenges with *S. pneumoniae* serotype 6B (10^4^ CFU/mouse). IL-4, TNF-α, and IFN-γ concentrations in serum and BAL samples were assessed on day 2 post-infection. The experiments were conducted in triplicate, with three mice per group (*n* = 9), and the results are presented as mean ± SD. Group distinctions are indicated by letters (*p* < 0.05).

When the levels of BAL cytokines were analyzed, both *Cp 090104* and BLPs significantly increased (*p* < 0.05) the concentrations of IL-4 and IFN-γ in the BAL when compared to controls. For TNF-α, there were no differences among groups after the infection with serotype 6B (Figure 5).

The mice immunized with PSPF and *Cp 090104* or BLPs showed enhanced concentrations of serum IFN-γ and TNF-α after the secondary pneumococcal challenge, while they did not modify the IL-4 levels in comparison to animals immunized with PSPF only (Figure 5). Similar results were obtained in the mice infected with *S. pneumoniae* serotype 19F.

## 4. Discussion

In our prior investigations, we demonstrated that nasal priming with *Cp 090104* or CP-derived BLPs influences the respiratory innate immune response [10,11,12]. This prompted us to examine whether this respiratory commensal bacterium’s innate immune modulation extends to adaptive immune responses. To explore this, we conducted a series of experiments involving the intranasal administration of *Cp 090104* or CP-derived BLPs to infant mice before *S. pneumoniae* exposure. This administration was carried out either independently or in combination with commercially available or experimental pneumococcal vaccines. Our systematic evaluation aimed to measure their effectiveness in triggering adaptive immune responses, encompassing both humoral and cellular components, and assessing their potential to enhance resistance against both primary and secondary pneumococcal infections. The results led us to draw three key conclusions, outlined as follows. (a) *Cp 090104* and CP-derived BLPs enhance the adaptive immune responses against pneumococcal infection, (b) *Cp 090104* and CP-derived BLPs enhance the adaptive immune responses induced by pneumococcal vaccines and boost resistance to primary pneumococcal infection, and (c) *Cp 090104* and CP-derived BLPs improve the adaptive immune responses induced by pneumococcal vaccines and enhance resistance to secondary pneumococcal infection.

***(a) Cp 090104 and CP-derived BLPs enhance the adaptive immune responses against pneumococcal infection***. Bacterial counts declined in the lungs and a reduction in lung injury parameters on day 7 post-infection was observed in the mice treated with *Cp 090104* or CP-derived BLPs before the pneumococcal infection. This observation aligns with our earlier findings, which focused on early infection assessed at day 2 post-challenge [11]. The protective effect can be credited to the bolstering of innate immunity and the heightened activation of antigen-presenting cells [11,13], resulting in a strengthening of both humoral and cellular adaptive immune responses. The impact of *Cp 090104* and CP-derived BLPs on adaptive immunity was apparent in the increased levels of antibodies, TNF-α, IFN-γ, and IL-4 found in the respiratory mucosa and bloodstream of the treated mice compared with the control mice.

Efficient protection against respiratory pathogens, such as pneumococci, depends on both mucosal and systemic antibodies. The secretory IgA is the principal antibody protecting the epithelial surfaces of the conducting airways while the lung parenchyma is defended by IgG [14,15]. Anti-pneumococcal capsular polysaccharide IgA antibodies were found to augment the complement-mediated bactericidal activity against bacteria [16,17]. Conversely, the mice deficient in IgA (IgA^−/−^) [18] and the animals lacking the receptor essential for IgA translocation to mucosal surfaces, the polymeric immunoglobulin receptor (pIgR^−/−^) [19], exhibited increased susceptibility to *S. pneumoniae* infection. This heightened susceptibility is attributed to their inability to generate mucosal IgA in response to pneumococcal vaccines, in contrast to wild-type animals. On the other hand, serum-neutralizing IgG antibodies against pneumococci had a key role in the control of their spread [17]. Dectin-2^−/−^ mice, which have an impaired ability to produce IgG in response to the PPV, had a significantly lower resistance to pneumococcal infection when compared to wild-type animals [20].

To evaluate some aspects of the cellular adaptive response, we measured TNF-α, IFN-γ and IL-4, which are key cytokines secreted by effector T cells. They play a significant role in activating macrophages and recruiting additional immune cells in response to the infections. The heightened levels of IFN-γ and IL-4 in BAL and serum samples from the mice treated with *Cp 090104* or CP-derived BLPs during pneumococcal infection indicated an enhancement in the activation of Th1 and Th2 cellular immune defenses. Previous research has demonstrated that comprehensive protection against *S. pneumoniae* needs a cellular immune response mediated by CD4^+^ T cells, particularly the Th1 subset that produces IFN-γ and IL-4 that support the production of antibodies [21,22].

After infection, we noted heightened TNF-α levels in the respiratory tract of mice pre-treated with *Cp 090104* or CP-derived BLPs, while in sera it was markedly lower than in the control group. Both beneficial and harmful effects have been described for TNF-α in the context of pneumococcal challenge, which depends on the time of infection and the compartment studied. Early studies showed that the administration of anti-TNF-α antibodies impaired neutrophil recruitment, bacterial clearance, and reduced survival in mice infected with *S. pneumoniae* [23]. On the other hand, uncontrolled inflammation and tissue damage facilitate pneumococcal pneumonia and invasion [24]. Excessive recruitment of monocytes and elevated TNF-α in old mice compared with young animals has been associated to impaired immunity and pathogen spread [25]. In addition, the elevated production of TNF-α in the later stages of *S. pneumoniae* infection has been associated with injury of the alveolar ultrastructure, edema, and an increased secretion of surfactant that can favor bacteria progression from the alveoli into the blood [26]. Furthermore, a strong release of TNF-α and IL-6 into the blood was associated with bacteremia. This background makes us speculate that the nasal priming of infant mice with *Cp 090104* or CP-derived BLPs would improve the local TNF-α response necessary for the clearance of the pathogen, which, together with the immune mechanisms mediated by antibodies and Th1/Th2 cells, would limit the dissemination of the pathogen. This would result in the decrease or complete prevention of pathogenic bacteria from reaching the blood, with the subsequent decrease in serum inflammatory cytokines such as TNF-α. It would be of value to characterize the variations over time of TNF-α and other inflammatory cytokines and chemokines. These kinetic studies could help understanding better the immunological changes induced by *Cp 090104* or CP-derived BLPs in the context of pneumococcal infection.

***(b) Cp 090104 and CP-derived BLPs enhance the adaptive immune responses induced by pneumococcal vaccines and boost resistance to primary pneumococcal infection.*** Our previous research showed that nasal priming with Cp 090104 and CP-derived BLPs enhances the effectiveness of the pneumococcal conjugate vaccine Prevenar^®^13 (PCV) by acting as mucosal adjuvants [13]. This study aimed to further investigate the adjuvant properties of Cp 090104 or CP-derived BLPs. Our findings demonstrated that nasal priming with PPV induced specific antibody production in the respiratory tract and blood, albeit at lower levels compared to nasal immunization with PCV [13] or PSPF. PPV primarily induces IgM antibodies due to its T-independent nature, similar to primary infections [27,28]. However, PPV’s efficacy in reducing pneumococcal cell counts upon infection challenge is lower than that of PCV [13] or PSPF, as evidenced by the higher pneumococcal counts in the lungs and blood of the PPV-immunized mice.

Interestingly, when *Cp 090104* or CP-derived BLPs were administered with PPV, the antibody production and pneumococcal count reduction were improved, indicating the adjuvant potential of *Cp 090104* or CP-derived BLPs. Although these treatments did not reach the same levels of antibody production and protection as PCV and PSPF, they enhanced the levels of TNF-α, IFN-γ, and IL-4 in the respiratory tract and blood post-challenge with pneumococci. These results align with recent studies showing that PPV can induce cellular immune responses, contrary to previous beliefs of its solely humoral immunity induction [28].

*Cp 090104*, like other Gram-positive bacteria, has a cell wall composed of glycolipids, lipoproteins, and proteins embedded in a peptidoglycan–arabinogalactan layer. Corynebacteria cell walls also feature sugar residues esterified by corynomycolic acids [29]. These bacterial components serve as microbe-associated molecular patterns (MAMPs), detectable using immune receptors such as TLR and NOD families in respiratory tract cells, including NOD1, NOD2, and TLR2, which sense peptidoglycan [30,31]. The interaction of TLR2 and NOD receptors with peptidoglycan induces cytokine production (TNF-α, IL-1β, IL-6), triggering antigen-presenting cell maturation and activation. BLP production from *Cp 090104* involves heat and acid treatment, increasing peptidoglycan exposure. This enhanced exposure likely improves TLR2 and/or NOD activation, enhancing BLPs’ adjuvant capacity compared to live bacteria. Future studies should identify the specific receptors activated by BLPs to enhance antigen presentation. Some researchers have also used other bacterial cell components such as flagellin as adjuvant. In this case, the proteinaceous nature of flagellin allows the creation of recombinant fusion proteins for vaccine antigens, which have shown efficacy in eliciting targeted immune responses [32,33]. In this work, we combined the use of a flagellin, included in the PSPF antigen, and BLP to stimulate TLR5 and TLR2, respectively. The adjuvant effect is the result of using two distinct PAMPs, which would explain why the immunization with BLP and PSPF was the most efficient to induce protection against pneumococcal infection.

Given that the immunomodulatory capacity of respiratory commensal bacteria is strain-specific [11], exploring if BLPs from other *C. pseudodiphtheriticum* strains share similar properties as *Cp 090104* is crucial for understanding their broader immunomodulatory potential.

***(c) Cp 090104 and CP-derived BLPs improve the adaptive immune responses induced by pneumococcal vaccines and enhance resistance to secondary pneumococcal infection.*** Secondary respiratory bacterial infections following primary viral infections can lead to significant morbidity and mortality in young individuals [34,35,36]. Clinical trials [37,38] and animal models [39,40] have demonstrated that the severity and mortality of these superinfections are also associated with enhanced lung tissue damage and pneumococcal dissemination into the bloodstream.

In a previous study, we found that nasal priming of mice with *Cp 090104* enhanced respiratory innate immunity, reduced lung pneumococcal counts, and prevented pathogen dissemination after RSV infection. Here, we assessed the effectiveness of nasal immunization with PSPF plus *Cp 090104* and CP-derived BLPs in protecting against secondary pneumococcal infection using a well-established superinfection model with poly(I:C) and *S. pneumoniae* [10]. Both live bacteria and BLPs improved infection resolution by reducing bacterial counts in the lungs. In this study, mice immunized with PSPF and PSPF plus *Cp 090104* showed reduced pneumococcal counts (serotype 6B), with the most significant reduction observed in mice receiving BLPs alongside PSPF, and the mice immunized with BLPs had 3.6 log pneumococcal counts (serotype 19F), which was significantly (*p* < 0.05) lower than that found in animals immunized with PSPF (5.6 log units) and PSPF plus *Cp 090104* (4.7 log units). These results could be explained using the enhanced levels of anti-pneumococcal antibodies and IFN-γ, both in the respiratory tract and blood, which would account for the more efficient control of *S. pneumoniae* secondary infection and for the remarkable effectiveness of BLPs.

While other studies have shown the effectiveness of PCV and PspA vaccination in preventing secondary pneumococcal infections, our work highlights the potential of mucosal immunizations with serotype-independent antigens, particularly in high-risk populations such as infants [41,42]. These findings contribute to the ongoing efforts to reduce morbidity and mortality from secondary pneumococcal infections, especially in scenarios involving complex viral–bacterial interactions and vulnerable populations.

## 5. Conclusions

In our various murine models, we assessed the ability of *Cp 090104* and CP-derived BLPs to modulate adaptive humoral and cellular immune responses against *S. pneumoniae*, both post-infection and post-nasal immunization, with two pneumococcal vaccines. Our findings indicate that *Cp 090104* and CP-derived BLPs elevated specific antibody and cytokine levels in the respiratory tract and blood, thereby enhancing the resistance of infant mice against challenges from different virulent pneumococcal serotypes. Consistent with our earlier studies on the innate immune response to *S. pneumoniae* [11], the effect of viable *Cp 090104* in the modulation of respiratory adaptive immune responses was slightly higher, a fact that is probably associated with the ability of the viable bacterium to colonize the respiratory mucosa and induce an optimal immunomodulatory effect. In addition, both *Cp 090104* and CP-derived BLPs increased the immunogenicity and protective capacity of two pneumococcal vaccines administered through the nasal route. Notably, the nasal priming with the PSPF T-dependent antigen co-administered with the respiratory commensal bacterium or the CP-derived BLPs efficiently stimulated humoral and cellular immunity and increased the resistance to primary pneumococcal challenge, as well as the secondary pneumococcal infection induced after the inflammatory lung damage mediated by the activation of TLR3. In these studies, BLPs presented a stronger effect than live bacteria. Utilizing BLPs or cellular components, such as the bacterial cell wall, rather than live bacteria presents potential avenues for immunomodulation, especially in at-risk populations where live microorganisms could present risks.

## Data Availability

All the data related to this project are presented here.
